# Clinical practice guidelines for the diagnosis and management of chronic lymphocytic leukemia in Saudi Arabia: consensus statement by an expert panel

**DOI:** 10.3389/fmed.2025.1719364

**Published:** 2026-01-14

**Authors:** Ghazi Alotaibi, Aamer Aleem, Ahmed Absi, Ahmed S. Alaskar, Feras Alfraih, Ayman Alhejazi, Ahlam Almasari, Musa Fares Alzahrani, Ibraheem Motabi, Riad El Fakih

**Affiliations:** 1 Department of Medicine, Division of Hematology/Oncology, King Saud University, Riyadh, Saudi Arabia; 2 King Abdulaziz Medical City, Jeddah, Saudi Arabia; 3 King Saud Bin Abdulaziz University for Health Sciences, Riyadh, Saudi Arabia; 4 King Faisal Specialist Hospital and Research Center, Jeddah, Saudi Arabia; 5 Department of Medicine, King Saud University, Riyadh, Saudi Arabia; 6 King Fahad Medical City, Riyadh, Saudi Arabia; 7 King Faisal Specialist Hospital and Research Center, Riyadh, Saudi Arabia; 8 College of Medicine, Alfaisal University, Riyadh, Saudi Arabia

**Keywords:** BCL2 inhibitor, BTK inhibitor, chronic lymphocytic leukemia, consensus, guideline, Saudi Arabia

## Abstract

Chronic lymphocytic leukemia (CLL) is the most common adult leukemia and represents a significant proportion of leukemias diagnosed in the Western countries but the true incidence and relative frequency of CLL compared with other leukemias in Saudi Arabia are not well defined. Survival outcomes in patients with CLL have improved substantially over time, largely due to the emergence of new, effective, and safer therapeutic options across different stages of the disease. There is a need for clear guidance in the optimal selection of patients for therapy, as well as guidance on therapeutic decision-making to achieve the best outcomes and reduce adverse events seen with the treatment. One of the important purposes of development of local guidelines is to unify the management approach of CLL and to facilitate and optimize early patient referral. This paper represents a focused review and consensus guideline from an expert panel in Saudi Arabia to guide diagnosis and management of CLL nationally. Recommendations are provided based on the available literature evidence and following consensus discussion among the expert panel, covering the diagnosis, staging, and management of CLL in the first line and relapsed/refractory settings.

## Introduction

Chronic lymphocytic leukemia (CLL), a disease of the older age, is characterized by the accumulation and clonal proliferation of mature but functionally incompetent B lymphocytes in the blood, bone marrow, spleen and lymph nodes (1, 2). In North America and Western/Central Europe, chronic lymphocytic leukemia (CLL) is one of the most common adult leukemias, with age-standardized incidence rates reported at approximately 3–5 per 100,000 persons per year in high-income countries such as the United States and Europe (3). The incidence of CLL varies according to the geography and demographics but the global burden has been increasing over time (4). Data from the Saudi Cancer Registry (SCR) Report shows that CLL represents 9% of all leukemias diagnosed, with the highest number of cases seen in the Central region (5). Survival of patients with CLL has shown considerable improvement over the past few decades, largely reflecting advances in treatment (6). It is essential that physicians are aware of advances in therapy and to effectively utilize the available therapeutic armamentarium in practice. This includes appropriate selection of therapeutic agents, timing of their use, and consideration of patient characteristics and profiles in therapeutic decision-making (7, 8).

The management of CLL in Saudi Arabia continues to face several challenges. Epidemiological and disease burden data remain limited; while the Saudi Cancer Registry provides annual reports, they often lack detailed information on treatment and outcomes. Some patients are not referred to tertiary care centers with the diagnostic capabilities required for proper risk stratification and treatment planning. Key molecular tests such as IGHV and TP53 mutation analysis are frequently unavailable in-house, necessitating overseas referral. The high cost of novel therapies can lead physicians to select suboptimal alternatives. Additionally, delays in regulatory approvals, limited drug availability, and the scarcity of clinical trials further restrict timely access to innovative treatments (9).

To date, there are no clinical guidelines tailored specifically to the diagnosis and management of CLL within the Saudi Arabian context. Considering recent therapeutic advances and the need for recommendations that consider local resources and regulatory frameworks, a panel of national experts was assembled to adapt and validate international CLL guidelines for implementation in Saudi Arabia.

### Methodology

A consensus panel of nine experienced CLL clinicians was selected, representing the major oncology centers across Saudi Arabia. An initial panel meeting was convened to establish the guideline’s objectives and methodology, ensuring alignment with the Saudi Health Council’s National Center for Evidence-Based Medicine (NCEBM) requirements for developing clinical practice guidelines (10). Three key questions were agreed as the priorities for focus in the Saudi Arabian setting:

*1. What are the recommended diagnostic and staging criteria for adult patients in Saudi Arabia with newly diagnosed CLL, including at diagnosis, at the time of therapy and upon relapse?*
*2. What are the recommended treatment strategies for adult patients in Saudi Arabia with newly diagnosed, relapsed, or refractory CLL?*
*3. What are the recommended follow-up and supportive care practices for adult patients in Saudi Arabia with CLL?*

A comprehensive literature search was conducted using online databases to identify relevant literature on the diagnosis and management of CLL in adults. The search strategy focused on peer-reviewed articles published in English between 1 January 2010 and December 2024 reporting human studies in adult populations (aged ≥ 18 years). Meta-analyses of randomized controlled trials (RCTs) and single-arm prospective studies were eligible for inclusion. Because of the rapid progress in the field, a literature review was carried out before the final revision of the manuscript and any new treatment approvals or practice changing studies published after the original search were also considered. The literature review was used as a basis to inform the development of consensus statements for the diagnosis and management of CLL. The International Workshop on CLL (iwCLL) guidelines for diagnosis, indications for treatment, response assessment, and supportive management of CLL (7) are well recognized and applicable for Saudi Arabia, therefore statements on CLL diagnosis, staging and prognostic assessment for Saudi Arabia were largely based on existing iwCLL recommendations.

Consensus was reached using a modified Delphi method. Prespecified cut-offs for recommendations were applied (statements with ≥70% agreement would be considered “strong” recommendations, those with 50%–70% agreement would be “weak” recommendations, and those with <50% agreements were not included) (Table 1 and Supplementary Table 1). Initial statements (thirty-six) developed based on the literature search were graded via an online survey completed by all members of the expert consensus panel. Panel members graded their level of agreement with each of the 36 statements. An optional free text comment box was provided for each question to justify or provide a rationale for the choice. Based on the findings of the initial survey (Supplementary Table 1), statements where consensus was not achieved were discussed and adjusted in a virtual meeting with all members of the consensus panel, followed by a second round of grading of the revised statements. Consensus recommendations were categorized as strong or weak depending on the criteria noted in Table 1. The following sections represent the findings of the panel consensus, supported by relevant literature and regional insights for Saudi Arabia.

**TABLE 1:** Criteria for classification of the strength of consensus recommendations, in line with Grading of Recommendations, Assessment, Development and Evaluation (GRADE) criteria.

Strong recommendation	Weak recommendation
Data derived from randomized controlled trials or meta-analysis	Data derived from retrospective, uncontrolled or observational studies
Consensus among ≥70% of respondents (graded as “agree” or “strongly agree”)	Consensus among 50%–69% of respondents (graded as “agree” or “strongly agree”)

## Diagnosis and staging of CLL

*1. Diagnosis*

Consensus recommendations for the diagnosis of CLL

1.1 Diagnosis of CLL requires the presence of ≥5 × 10^9^/L clonal B-lymphocytes in the peripheral blood, sustained for at least 3 months. (STRONG)
1.2 The clonality of B lymphocytes should be confirmed by demonstrating immunoglobulin light chain restriction using flow cytometry. A panel of CD19, CD5, CD20, CD23, κ, and λ is usually sufficient to establish the diagnosis. (STRONG)
1.3 Molecular genetics, mutational status of IGHV and variable heavy stereotypes, immunophenotypic markers, serum markers and marrow examination are not essential to diagnose CLL but are important to predict the prognosis and/or assess the tumor burden. (STRONG)

The diagnosis of CLL is established by evaluating the blood smear and the immunophenotype of the circulating lymphocytes, as well as the use of genetic features of the circulating lymphoid cells in some patients. Peripheral blood smear assessment is used to evaluate the morphology and the number of B lymphocytes. Consistent with international guidelines published by the iwCLL (7), the expert panel agreed that the diagnosis of CLL requires the presence of ≥5 × 10^9^/L clonal B lymphocytes in the peripheral blood, which is sustained for at least 3 months (Recommendation 1.1).

To confirm the clonality of B lymphocytes, flow cytometry and immunophenotyping using a defined panel of markers is needed (Recommendation 1.2). The immunophenotyping of CLL cells is valuable in supporting the diagnosis; a harmonization project of the European Research Initiative on CLL (ERIC) and the European Society for Clinical Cell Analysis (ESCCA) confirmed that a panel of CD19, CD5, CD20, CD23, κ, and λ light chains is typically sufficient to diagnose CLL (11). In atypical CLL, cells may show stronger CD20 expression or brighter surface light chain restriction, which can overlap with other B-cell neoplasms. In borderline cases, refinement of the diagnosis may be achieved through additional marker evaluation, including CD43, CD79b, CD81, CD200, CD10, or ROR1 (11). Furthermore, cytogenetic evaluation using FISH, particularly for t(11;14)(q13;q32), is recommended to distinguish CLL from mantle cell lymphoma in atypical presentations.

Additional tests are not considered to be essential for the diagnosis of CLL but add value in prognosis or tumor burden assessment (Recommendation 1.3). These tests include molecular genetic testing using fluorescence *in situ* hybridization (FISH) of peripheral blood lymphocytes and conventional karyotyping approaches (12). These strategies may have value in differentiating CLL from other lymphoproliferative diseases and can identify genetic abnormalities that may inform treatment decisions. For instance, deletions of the long arm of chromosome 11 (del11q) and the short arm of chromosome 17 (del17p) and mutations in the *TP53* gene are associated with poorer prognosis and resistance to standard chemoimmunotherapy (CIT) (12–15). TP53 aberrations (Del17p and/or *TP53* mutation) carry a poor response to conventional CIT but better response rates with non-chemotherapeutic agents (13). Progression-free survival (PFS) and overall survival (OS) are similar in patients with del17p or *TP53* mutations (16). Therefore, genetic analysis, particularly for *TP53* aberrations, has prognostic value and may guide therapeutic decision-making. Re-assessment of genetic abnormalities should be completed prior to initiating therapy and should be repeated before initiating new lines of therapy due to the risk of acquired genetic abnormalities over the disease course (17).

Other recommended assessments include the mutational status of IGHV genes, identification of stereotyped B-cell receptor IGs, immunophenotypic markers, serum markers and marrow examination, as noted in iwCLL guidelines (7). Patients with unmutated IGHV show poorer survival from the time of diagnosis compared with patients with mutated IGHV (18–20). IGHV mutational status is a recognized prognostic factor in CLL included in the disease-specific International Prognostic Index (CLL-IPI) (21). Different subsets of stereotyped B-cell receptor IGs demonstrate distinct characteristics, with prognostic implications, and may have value in patient risk stratification (22). Some evidence also supports a prognostic value for immunophenotypic markers, such as ZAP-70, CD38 and CD49b expression as well as serum markers including β_2_-microglobulin (23–25). Bone marrow examination may have value in attributing cytopenias to leukemic infiltration of the marrow versus other causes and in clarifying treatment-related versus disease-related cytopenias during therapy (7).

*2. Staging of CLL*

Consensus recommendations for CLL staging

2.1 The modified Rai classification defines low-risk disease as lymphocytosis with leukemia cells in the blood and/or marrow. Patients with peripheral blood lymphocytosis, enlarged lymph nodes in any site, and splenomegaly and/or hepatomegaly (lymph nodes being palpable or not) are defined as having intermediate-risk disease. Patients with disease-related anemia or thrombocytopenia (regardless of presence or absence of above features) are categorized as having high-risk disease. (STRONG)
2.2 The Binet staging system is based on the number of involved lymphoid areas (defined by the presence of enlarged lymph nodes ≥ 1 cm in diameter or organomegaly) and presence of anemia or thrombocytopenia. Areas of involvement considered for staging include head and neck, the Waldeyer ring, axillae, groins, superficial femoral, palpable spleen, and palpable liver. Stage A is defined as Hb ≥ 10 g/dL and platelets ≥ 100 × 10^9^/L and ≤2 areas involved. Stage B is defined as Hb ≥ 10 g/dL and platelets ≥ 100 × 10^9^/L and ≥3 areas involved. Stage C is considered as Hb < 10 g/dL and/or a platelet count < 100 × 10^9^/L. (STRONG)
2.3 The CLL International Prognostic Index (CLL-IPI) includes clinical stage, age, IGHV mutational status, serum b2-microglobulin, and presence of del17p and/or TP53 mutations and can be used to identify high-risk patients. (STRONG)
2.4 The Cumulative Illness Rating Scale (CIRS) may be used to assess comorbidities in CLL, although other scores to identify unfit patients are available. (STRONG)

Staging of CLL is important to stratify patients according to disease risk. Two staging systems are commonly used in practice: the Binet system (26) and the modified Rai classification (27) (Table 2). Both systems rely on physical examination and laboratory data, without the need for imaging studies, and are inexpensive and easy to implement in practice. The modified Rai classification stratifies patients into low-, intermediate- or high-risk groups (Recommendation 2.1) and the Binet staging system stratifies patients into stages A, B or C (Recommendation 2.2). Although no specific preference is mandated, the modified Rai classification is more frequently used in Saudi Arabia, likely due to better clinical familiarity and comfort with its application.

**TABLE 2:** Comparison of the Binet staging system and the modified Rai classification for the staging of CLL (8).

Binet staging system	Modified Rai classification
Stage A <3 areas* of lymphadenopathy	Low-risk disease Lymphocytosis only
Stage B ≥3 areas* of lymphadenopathy	Intermediate risk disease Lymphocytosis with lymphadenopathy ± splenomegaly or hepatomegaly
Stage C Hb ≤ 10 g/dL and/or platelets < 100 × 10^9^/L	High-risk disease Lymphocytosis with anemia (Hb < 1 g/dL) or thrombocytopenia (platelets < 100 × 10^9^/L)

Hb, hemoglobin.

*Areas of lymphadenopathy assessed are head and neck (including the Waldeyer ring), axillae, groins, palpable spleen or palpable liver.

The prognostic value of staging systems is enhanced with the inclusion of key biomarkers, as indicated in the previous section. This includes prognostic factors such as del17p or TP53 mutations, IGHV mutational status and serum β_2_-microglobulin levels (7). Prognostic scoring systems incorporating clinical and para clinical markers have been validated, such as the CLL-IPI (21). These scores are particularly valuable in identifying high-risk patients (Recommendation 2.3). Scoring systems such as the Cumulative Illness Rating Scale (CIRS) has been recognized in characterizing patients with comorbidities (7, 28, 29), especially in the context of clinical trial eligibility (Recommendation 2.4). While the advent of targeted therapies has improved outcomes, particularly in patients with high-risk features, prognostic assessment remains important for guiding optimal treatment selection especially in the frontline setting.

## Treatment of CLL

The CLL expert panel recommends initiation of treatment in CLL based on the presence of active or symptomatic disease, as defined by the iwCLL guidelines (7). Indications for therapy include progressive bone marrow failure, significant or symptomatic splenomegaly and/or lymphadenopathy, and steroid-refractory autoimmune cytopenias. Other triggers include symptomatic or functionally relevant extra-nodal involvement, as well as disease-related symptoms such as weight loss, marked fatigue, unexplained fever, and night sweats. Progressive lymphocytosis or a lymphocyte doubling time of less than 6 months is suggestive of disease progression and warrants close monitoring, though it is not in itself an automatic indication for initiating therapy. Conversely, isolated findings such as hypogammaglobulinemia, paraproteinemia, or an elevated absolute lymphocyte count should not be used as sole criteria for starting treatment (7).

Following identification of patients eligible for treatment, selection of first-line treatment options is crucial for optimal outcomes. Therapeutic options for CLL include Bruton’s tyrosine kinase inhibitors (BTKi), B-cell lymphoma-2 inhibitors (BCL2i), monoclonal antibodies (e.g., anti-CD20), phosphoinositide 3-kinase (PI3K) inhibitors and chemoimmunotherapy (CIT). The relative value of these agents and their suitability in the first-line setting depends on the prognostic factors and the clinical characteristics of patients. The following sections elaborate on treatment-specific guidance for specific patient groups, based on symptoms, fitness, age and genetic characteristics.

*3. Early-stage asymptomatic patients*

Consensus recommendation for early-stage asymptomatic patients with CLL

3.1 A “watch and wait” strategy is recommended in patients with early asymptomatic disease. (STRONG)

Patients with early-stage, asymptomatic CLL (Rai 0 or Binet A classification) have no survival benefit with early treatment (30, 31). More recently, data on early intervention using the CIT with fludarabine, cyclophosphamide, and rituximab (FCR) versus observation showed no OS benefit over 5 years (32). Early use of ibrutinib was found to improve event-free survival compared with observation in patients with Binet stage A disease at high-risk of progression (hazard ratio 0.25; 95% confidence interval, 0.14–0.43; *P* < 0.0001) although this study did not show an overall survival (OS) benefit (33). Therefore, asymptomatic, early-stage disease is not considered an indication for active treatment, and it is recommended that a “watch and wait” strategy is employed to monitor for disease progression or symptoms (Recommendation 3.1).

*4. Symptomatic CLL patients who are young and fit without del17p or TP53 mutations*

Consensus recommendations for symptomatic patients (young, fit and low risk)

4.1 Chemotherapy is no longer recommended, given the overall survival benefit with ibrutinib + rituximab versus FCR in the E1912 trial and the risk of secondary malignancies with FCR. (STRONG)
4.2 BTK inhibitor monotherapy is one of the options for patients with CLL without del17p mutation or TP53 aberrations who require treatment. (STRONG)
4.3 Venetoclax + Obinutuzumab is recommended as a time-limited therapy. (STRONG)
4.4 Ibrutinib + Venetoclax is another fixed duration treatment option in patients younger then 60 years with no underlying comorbidities. (STRONG)
4.5 Minimal residual disease (MRD) assessment is not typically used in clinical practice, but MRD-guided therapy may become a feasible future strategy. (STRONG)

When patients present with symptomatic/active CLL, first-line therapy in those without del17p or *TP53* mutations and who are young and fit, was formerly based on the FCR regimen (34). Studies have shown a high progression free survival (PFS) with FCR therapy compared with alternative CIT regimens, suggesting long-term remission can be achieved with FCR regimen in these patients (18, 35). However, recent data have challenged the use of FCR in this group as the preferred first-line option (36–38).

The ECOG 1912 trial (36, 37) demonstrated that ibrutinib plus rituximab is superior to FCR in fit, young (<70 years) patients. At a median follow-up of 33.6 months, PFS and OS were superior with ibrutinib + rituximab compared with FCR (89.4% versus 72.9%, *P* < 0.001; and 98.8% versus 91.5%, *P* < 0.001, respectively). The subgroup analysis of this trial showed that the greatest benefit was seen in patients with unmutated IGHV, although PFS benefits were also evident in the mutated IGHV subgroup. The phase 3 FLAIR trial (38) also showed superior PFS with ibrutinib + rituximab therapy compared with FCR over a 52.7-months follow-up period. In addition, FCR is associated with an increased risk of secondary malignancies in the CLL population (18, 39). Consequently, chemotherapy is no longer considered a recommended first-line treatment in this population (Recommendation 4.1).

Three BTKi are available in Saudi Arabia for the treatment of CLL; ibrutinib, acalabrutinib, and zanubrutinib. Ibrutinib was the first agent approved for the treatment of CLL, based on initial data supporting ibrutinib monotherapy as a first-line option compared with chlorambucil monotherapy (40). As chlorambucil is no longer considered a first-line treatment option for CLL, further studies have been conducted to support the use of ibrutinib as monotherapy or in combination with other agents. The ALLIANCE A041202 study showed that patients without TP53 mutation had significantly improved PFS with ibrutinib or ibrutinib + rituximab therapy compared with CIT with bendamustine + rituximab (41). In this study, there was no difference between the ibrutinib and ibrutinib + rituximab arms, suggesting that addition of rituximab may not enhance efficacy of BTKi monotherapy, a conclusion supported by another RCT comparing ibrutinib and ibrutinib + rituximab in first-line CLL therapy (42). Studies of second-generation BTKi, with better target specificity than ibrutinib, have confirmed the value of BTKi monotherapy in young, fit patients without TP53 mutations. The ELEVATE-TN study reported that acalabrutinib, either as monotherapy or in combination with the anti-CD20 monoclonal antibody obinutuzumab provided superior PFS compared with chlorambucil + obinutuzumab (43). This combination is reflected in recent National Comprehensive Cancer Network (NCCN) guidelines for CLL treatment (44). The SEQUOIA study demonstrated the superiority of zanubrutinib monotherapy versus bendamustine + rituximab in patients without del17p (45). Together, these studies support the use of BTKi monotherapy in young, fit patients without TP53 mutations or del17p (Recommendation 4.2).

Recent NCCN guidelines moved ibrutinib to a “less preferred” treatment option versus second-generation BTKi (44), but the expert panel agreed to retain ibrutinib as a reasonable option in clinical practice for Saudi Arabia based on the availability of long-term data depicting the efficacy and safety of ibrutinib, familiarity of physicians with the drug, and the emergence of generics and its cost implications.

As the BTKi are given in a continuous fashion, other therapeutic regimens may be considered in young, fit patients without *TP53* mutations or del17p. These include venetoclax + obinutuzumab and ibrutinib + venetoclax. The CLL14 trial and CLL13 (46, 47) demonstrated the efficacy of venetoclax + obinutuzumab as a fixed-duration therapy, with superior PFS compared to chlorambucil-based regimens. CLL14 compared fixed-duration venetoclax + obinutuzumab with chlorambucil + obinutuzumab in 432 treatment-naïve patients with only 8% having del17p mutations. At a median follow-up of 52 months, the estimated 4-years PFS was 74% vs. 35% (*P* < 0.0001) with venetoclax + obinutuzumab compared with chlorambucil + obinutuzumab, respectively. In addition, the CLL13 trial suggested patients with CLL had a superior PFS when venetoclax was combined with obinutuzumab rather than rituximab (47). Therefore, venetoclax + obinutuzumab is recommended in this population as a fixed-duration therapy as one of the options (Recommendation 4.3).

The fixed-duration combination of ibrutinib + venetoclax is another potential treatment option in this patient group. The rationale for this approach is based on the findings from several studies (48, 49), including the GLOW phase 3 RCT comparing ibrutinib + venetoclax with chlorambucil + obinutuzumab in 211 treatment-naive patients, excluding those with del17p (50). The estimated 30-months PFS was 80% and 36% with ibrutinib + venetoclax versus chlorambucil + obinutuzumab, respectively, with longer-term follow-up supporting these findings (49). Of note, the GLOW trial included an older patient cohort with frequent comorbidities and a higher risk of grade 3 and 4 adverse events associated with the ibrutinib + venetoclax regimen was noted, including infections and atrial fibrillation. Therefore, given the significant toxicity seen with this regimen, it may be avoided in older patients and offered only to younger, fit patients with previously untreated CLL (Recommendation 4.4). In addition, the AMPLIFY trial (51) evaluated acalabrutinib combined with venetoclax, with or without obinutuzumab, in both treatment-naïve and relapsed/refractory CLL. This study demonstrated high rates of deep responses and undetectable minimal residual disease, supporting its potential as an effective combination therapy option in fit patients, complementing existing BTKi- and venetoclax-based strategies.

Assessment of minimal residual disease (MRD) at the end of a fixed-duration treatment period may have prognostic value (44), although achieving undetectable MRD does not appear to have a prognostic impact with continuous BTKi regimens (50). Ongoing trials exploring different combinations of fixed-duration therapies with continuous BTKi therapy as monotherapy or in combination may clarify the value of MRD assessment and its role in practice. Therefore, while not routinely assessed in practice, use of MRD to guide treatment may be needed in the future (Recommendation 4.5).

*5. Symptomatic CLL patients with del17p, TP53 mutations, or both*

Consensus recommendations for symptomatic CLL with del17p, TP53 mutations, or both

5.1 Continuous BTKi therapy is recommended in patients with CLL who have del17p and/or TP53 aberrations. (STRONG)
5.2 Venetoclax–obinutuzumab is one of the options for patients who choose a time-limited treatment. (STRONG)
5.3 Venetoclax-acalabrutinib (preferred) or venetoclax-ibrutinib (less preferred) are a time limited treatment options for these patients (STRONG)

We recommend BTKi as the standard of care in this high risk CLL population. This recommendation is supported by a pooled analysis of four clinical trials of ibrutinib with or without rituximab in patients with *TP53* disruption (*n* = 89), where the 4-years PFS was 79% and the 4-years OS was 88% (52), which compares favorably with standard CIT-based regimens in patients with *TP53* disruptions. Therefore, BTKi are a key treatment option in patients with *TP53* aberrations (53, 54) (Recommendation 5.1).

Fixed-duration venetoclax + obinutuzumab was associated with 24-months PFS of 88.2% as compared to 64.1% in the chlorambucil-obinutuzumab arm in a phase 3 trial of Patients with CLL and co-existing conditions (55). This benefit was also observed in patients with *TP53* deletion/mutation, or both and in patients with unmutated immunoglobulin heavy-chain genes (56). Therefore, the combination of venetoclax + obinutuzumab is a potential fixed duration option in this group of patients (Recommendation 5.2). For patients who prefer fixed duration BTKi containing therapy, the group prefers venetoclax-acalabrutinib over venetoclax-ibrutinib combinations as a time-limited treatment option (56, 57).

*6. Symptomatic CLL patients who are older and/or unfit without del17p or TP53 mutations*

Consensus recommendations for symptomatic CLL patients who are older and/or unfit without del17p or TP53 mutations

6.1 Venetoclax + obinutuzumab is recommended as a time-limited therapy for these patients. (STRONG)
6.2 BTKi (ibrutinib, acalabrutinib, or zanubrutinib) are recommended as continuous therapy. (STRONG)
6.3 Second-generation BTKi (e.g., acalabrutinib and zanubrutinib) have a more favorable toxicity profile in patients with comorbidities, but ibrutinib remains a reasonable option given the availability of long-term data and the experience of physicians in managing ibrutinib therapy. (STRONG)
6.4 There is no added benefit of rituximab in combination with BTKi. (STRONG)
6.5 Obinutuzumab combined with BTKi is associated with additional benefit. (WEAK)
6.6 Chlorambucil + obinutuzumab is not apreferred option in patients who are not fit for therapy. (STRONG)

Older and unfit patients represent a substantial proportion of those requiring frontline therapy for CLL. In this population, selecting a regimen with a favorable safety and efficacy profile is essential. One such fixed-duration option is venetoclax plus obinutuzumab (Recommendation 6.1). In the CLL14 trial, this regimen was associated with a 4-years progression-free survival (PFS) of 74% compared with 35% for chlorambucil plus obinutuzumab (*P* < 0.0001) in patients with a median age of 72 years and a median CIRS score of 8 (58). This combination also demonstrated higher rates of undetectable MRD and longer PFS compared with venetoclax plus rituximab in indirect comparisons (47).

Continuous therapy with BTKi may be considered a second preferred treatment option in this patient group, as demonstrated in the ALLIANCE A041202 (41) and RESONATE-2 trials (40) Both trials reported favorable long-term PFS with ibrutinib in older patients (≥65 years) and those with comorbidities (69% CIRS score > 6 in the RESONATE-2 trial) (Recommendation 6.2).

The toxicity profile of second-generation BTKi (acalabrutinib and zanubrutinib) may suggest their preferred use over ibrutinib in older patients and patients with comorbidities (59, 60). However, ibrutinib is still considered a valid treatment option in this group based on long-term efficacy and safety data, as well as availability of a generic options and longer physician experience in Saudi Arabia (Recommendation 6.3).

As noted in the ALLIANCE A041202 study, patients without *TP53* mutations (including older patients) had significantly improved PFS with ibrutinib regardless of the addition of rituximab (41). Similar results were reported from other studies, suggesting that addition of rituximab does not add to BTKi monotherapy efficacy in this context (42, 43). Therefore, the addition of rituximab to BTKi is not necessary in this patient group (Recommendation 6.4). The ELEVATE-TN trial (43), however, showed some benefits (particularly expediting response) from adding obinutuzumab to BTKi in older/unfit patients with autoimmune conditions or glomerulonephritis. However, more data are needed to potentially support this combination in the future (Recommendation 6.5).

The combination of chlorambucil with obinutuzumab is not a recommended option anymore based on the GLOW trial results where chlorambucil + obinutuzumab was associated with a lower PFS and lower undetectable MRD rate compared with ibrutinib + venetoclax (61). Similarly, the CLL14 trial showed that chlorambucil + obinutuzumab was inferior to venetoclax + obinutuzumab in patients with comorbidities after a follow up of 52-months (58). Therefore, the combination of chlorambucil and obinutuzumab is not considered a preferred option in this patient group (Recommendation 6.6).

*7. Relapsed/refractory (R/R) disease*

Consensus recommendations for relapsed/refractory CLL treatment

7.1 Chemoimmunotherapy is not recommended for relapsed or refractory CLL. (STRONG)
7.2 BTKi or venetoclax in combination with rituximab are recommended in R/R disease regardless of the mutation status, depending on the first-line regimen used. (STRONG)
7.3 BTKi may be preferred over venetoclax with rituximab in patients with TP53 aberrations. (STRONG)
7.4 A BTKi in combination with venetoclax may be considered in all patients with R/R disease regardless of mutation status. (STRONG)
7.5 If therapy is sequenced, venetoclax is preferred after a BTKi. (STRONG)
7.6 Pirtobrutinib should be considered in patients refractory to a covalent BTKi and a BCL2 inhibitor. (STRONG)
7.7 PI3K inhibitors have efficacy in R/R CLL, but their value is limited by their safety profile. (STRONG)
7.8 Clinical trials are recommended for patients with relapsed or refractory CLL who are refractory to both BTK and BCL2 inhibitors. (STRONG)
7.9 Beyond two lines of therapy, CAR-T therapy should be considered in patients with rapidly progressing disease. (STRONG)

Relapsed CLL is defined as disease progression after achieving partial or complete remission for 6 months after therapy. Refractory CLL is defined as non-response to therapy, progression while on treatment or progression within 6 months of a time-limited therapy (7). CIT is not recommended in relapsed/refractory (R/R) CLL (Recommendation 7.1), given that novel therapies show superior efficacy and better safety in this patient group. In patients who have previously received CIT only, the use of BTKi or BCL2i in combination with anti-CD20 monoclonal antibodies (rituximab) should be considered (Recommendation 7.2). In patients with previous exposure to one novel therapy, use of an alternative novel therapy should be considered. Other options may include re-treatment with fixed-duration combinations (e.g., venetoclax + rituximab) with the same combination if significant benefit was achieved with the first course of therapy. The panel recommends that in case of BTK intolerance, an alternative BTKi can be used.

Recent trials in the R/R CLL setting suggest that second-generation BTKi (acalabrutinib and zanubrutinib) have a consistent safety and efficacy profile compared with ibrutinib. The ALPINE trial (60) reported that zanubrutinib was associated with a PFS of 78.4% at 24 months compared with 65.9% with ibrutinib in patients with R/R CLL (*n* = 652). In the ELEVATE-RR study (59), acalabrutinib was non-inferior to ibrutinib in patients with R/R CLL with 17p and 11q deletions (*n* = 533), with a median PFS of 38.4 months in both arms. Together, these studies suggest the non-inferiority of second-generation BTKi compared with ibrutinib. In terms of safety, both studies reported lower toxicity rates, reduced incidence of atrial fibrillation and fewer treatment discontinuations (due to adverse events) with second-generation BTKi versus ibrutinib (62, 63). Despite the potentially more favorable safety profile of newer-generation BTKi, the expert panel supports the use of ibrutinib in Saudi Arabia due to its wide availability, long-term clinical data, extensive experience among clinicians, and the accessibility of generic formulations.

There is some evidence to suggest that BTKi may be preferred over venetoclax + rituximab in patients with *TP53* aberrations (Recommendation 7.3). This is based on studies in the first-line treatment setting, where it is recognized that BTKi therapy is a standard of care for patients with *TP53* mutations. In the R/R CLL setting, *TP53* mutation analysis should be repeated prior to therapeutic decision-making due to the risk of mutation-emergence on treatment (7). A BTKi in combination with venetoclax may be considered in all patients with R/R CLL disease, regardless of mutation status (Recommendation 7.4). This recommendation is based on evidence from a range of studies supporting the clinical benefits of ibrutinib in combination with venetoclax in the R/R CLL setting, including CLARITY (64), VISION (65) and IMPROVE trials (66). Similarly, data from the SEQUOIA study (53) demonstrated the efficacy and safety of zanubrutinib + venetoclax, which may have relevance in the R/R CLL setting. These studies cover a range of patients, including those with and without high-risk mutations, different age groups and different levels of fitness, suggesting that this may be applicable across most patients with R/R CLL.

Therapy sequencing in R/R CLL remains an area of active discussion, given the absence of randomized trials comparing BTK inhibitor monotherapy or combinations followed by venetoclax plus rituximab, or vice versa (6). In the MURANO study, ibrutinib was used as the first subsequent therapy in 12 patients after progression on venetoclax–rituximab, with all 10 evaluable patients achieving a response (100%) (67). Conversely, sequencing BTK inhibitors before venetoclax showed an overall response rate (ORR) of 65% and a median PFS of 24.7 months in an open-label phase 2 trial involving 91 patients (68).

Due to the lack of head-to-head trials comparing BTK and BCL2 inhibitors in R/R CLL, treatment decisions should be individualized, considering patient characteristics, treatment goals, comorbidities, concurrent medications, prior therapies, and access to drugs or clinical trials. Sequencing should also consider whether prior treatments were discontinued due to resistance or intolerance. Based on current evidence, the expert panel recommends initiating therapy with a BTKi followed by venetoclax, given the robust response rates observed with venetoclax after BTKi failure (Recommendation 7.5).

Pirtobrutinib, a non-covalent BTK inhibitor, is approved by the Saudi FDA for R/R CLL after at least two prior lines of therapy, including a BTKi and a BCL2i, based on the BRUIN phase I–II study (69, 70). The study reported an ORR of 73% [ORR; 82% including partial response with lymphocytosis (PR-L)] and a median PFS of 20 months in BTKi-pretreated patients (*n* = 247), with an 18-months OSR of 81%. In patients previously treated with both BTKi and venetoclax (*n* = 100), ORR was 70% (79% including PR-L) with a 17-months PFS. ORR remained high regardless of prior BCL2i exposure (83% naïve vs. 80% exposed), though PFS was longer in the naïve group (23 vs. 16 months) (62, 63). The phase III BRUIN CLL-321 study demonstrated superior PFS with pirtobrutinib (HR, 0.55; *P* = 0.0007) compared with bendamustine or idelalisib plus rituximab in BTKi-pretreated patients, including those with prior venetoclax (HR, 0.54) or TP53 mutation/del(17p) (HR, 0.52) (70).

The Panel recommends pirtobrutinib as a preferred treatment option for patients with R/R CLL after at least two prior lines of therapy, including a BTKi and a BCL2i (venetoclax), due to its efficacy and favorable toxicity profile in double-refractory CLL (Recommendation 7.6).

In R/R CLL, PI3K inhibitors have shown efficacy but are limited by significant toxicity. In the phase III DUO study, duvelisib monotherapy achieved an OR rate of 74% and a median PFS of 13.3 months versus 9.9 months with ofatumumab (ORR 45%) (PMID:30287523). In a pivotal phase III trial, idelalisib combined with rituximab resulted in an ORR of 81% versus 13% for placebo plus rituximab, with median PFS not reached in the idelalisib arm and only 5.5 months in the control group (71). Long–term follow–up data reported a median PFS of 20.3 months and an ORR of 85.5% for idelalisib–rituximab (72). However, PI3K inhibitors are associated with high rates of grade ≥ 3 adverse events; neutropenia (∼34%), diarrhea/colitis (∼16%–17%), and pneumonitis (∼6%), which constrain their clinical utility. Based on these concerns, the expert panel recommends reserving PI3K inhibitor use for carefully selected patients (Recommendation 7.7).

Although, availability of clinical trials is limited in Saudi Arabia, the panel recommends participation in clinical trials, whenever possible, for patients with R/R CLL who are refractory to multiple pathway inhibitors (Recommendation 7.8).

CD19-directed CAR T-cell therapy has shown significant efficacy in R/R CLL, with trials involving over 200 patients demonstrating ORR of 44%–74% and complete response (CR) rates of 8%–30%, with durable remissions in select patients (73, 74). The TRANSCEND CLL-004 study (*n* = 117) of lisocabtagene maraleucel (liso-cel) reported an ORR of 44%–64% (monotherapy) and 86% (with ibrutinib), CR rates of 20%–25%, and a median PFS of 12 months for monotherapy, with grade 3–4 cytokine release syndrome (CRS) in 7%–9% and neurotoxicity in 13%–20% of patients (73). Liso-cel received U.S. FDA approval in March 2024 for R/R CLL/SLL after failure of at least two prior lines, including a BTKi and a BCL2i. Pending approval and availability in Saudi Arabia, the expert Panel recommends liso-cel for R/R CLL patients with rapidly progressing disease post two lines of therapy, prioritizing those with suitable performance status and manageable toxicity risks (Recommendation 7.9).

*8. Allogenic Hematopoietic stem cell transplantation (HSCT)*

Consensus recommendations on HSCT in patients with CLL

8.1 Allogeneic HSCT from a matched related or unrelated donor can be considered in younger, fit patients with high-risk CLL who have failed at least one pathway inhibitor. (WEAK)
8.2 Patients should be referred to a transplant center for consultation once their CLL has proved refractory to at least one pathway inhibitor. (WEAK)
8.3 Older patients should generally not be considered for allogeneic HSCT due to high mortality rates. (STRONG)

Hematopoietic stem cell transplantation is infrequently used in CLL due to the disease’s typically slow progression and improved outcomes with novel therapies (75). HSCT may be an option for high-risk CLL patients who have progressed after treatment with a pathway inhibitor (7). A small subset of patients who have failed BTKi, BCL2 inhibitors, and CAR-T therapy may also benefit, although careful patient selection is critical to maximize HSCT outcomes. Advanced age and poor performance status increase the risk of non-relapse mortality, indicating that HSCT should be reserved for younger, physically fit patients with an estimated one-in-three likelihood of achieving long-term disease-free survival (76). Accordingly, the expert panel recommends considering allogeneic HSCT for younger, fit, high-risk CLL patients who have failed a pathway inhibitor (Recommendation 8.1). Patients meeting these criteria should be referred early to a transplant center for evaluation (Recommendation 8.2). Due to elevated mortality risks in older patients, HSCT is generally not recommended for them, especially when alternative treatments are available (Recommendation 8.3). These recommendations align with current guidelines on HSCT in CLL (7, 44).

*9. Supportive care*

Consensus recommendations for supportive care in patients with CLL

9.1 Patients with CLL should receive annual influenza vaccination, and COVID-19, pneumococcal, and Herpes zoster immunizations according to the recommended schedules. Live vaccines should not be given during periods of immunosuppression from chemotherapy or immunotherapy. (STRONG)
9.2 Antimicrobial prophylaxis requirements vary by therapeutic regimen, but vigilant observation and early initiation of treatment are warranted if symptoms of infection develop. (STRONG)
9.3 Prophylactic intravenous immunoglobulin (IVIG) is indicated for patients with hypogammaglobulinemia who have recurrent serious infections. (STRONG)
9.4 CLL patients should be screened for HBV prior to starting therapy with anti-CD20 mAb-containing regimens, idelalisib, and purine analogs. (STRONG) HBV carriers should start prophylactic antiviral therapy with entecavir before initiating these treatments. (STRONG)
9.5 Consider referring patients for a dermatology examination once a year. (WEAK)

Regardless of the treatment regimen, patients with CLL should have adequate supportive care to ensure optimal outcomes and wellbeing. Infections are a frequent challenge and prevention of infections is a key strategy to prevent adverse outcomes (77). Vaccines against influenza, COVID-19, pneumococcal disease and herpes zoster may be recommended due to the severity of these infections, particularly in immunocompromised patients (78). Where possible, live vaccinations should be avoided during periods of immunosuppression, ideally given prior to initiation of treatment (78). The expert panel recommends routine vaccination in this patient group as a key feature of supportive care (Recommendation 9.1).

Other strategies to mitigate infection risk include the use of antimicrobial prophylaxis and prophylactic intravenous immunoglobin (IVIG) therapy. To date, there are no RCTs evaluating the use of antimicrobial prophylaxis in CLL patients, with data derived from anecdotal reports or regimens applied in trials. Requirements for prophylaxis may therefore vary by specific treatment regimen and clinical features of the patient (79). Physicians should monitor for early signs of infection and initiate prompt antimicrobial treatment (Recommendation 9.2). While prophylactic IVIG may reduce bacterial infections and delay their onset during CLL therapy (80), it has not shown survival or quality-of-life benefits and is not cost-effective (81–83). Therefore, IVIG is not routinely recommended but should only be considered in patients with hypogammaglobulinemia and recurrent serious infections (Recommendation 9.3).

Hepatitis B virus (HBV) reactivation can occur in HBV carriers treated with chemotherapy and/or immunotherapy. CLL patients should be screened for HBV prior to starting therapy with anti-CD20 mAb-containing regimens, idelalisib, and purine analogs. HBV carriers should receive prophylactic antiviral therapy with entecavir before starting one of the above treatments (84). Antiviral prophylaxis should be continued for 12 months after completion of CLL treatment (85) (Recommendation 9.4). HBV reactivation has been reported in occasional patients receiving ibrutinib. Because of the rarity of the HBV reactivation in CLL patients receiving ibrutinib, currently it is not clear whether all CLL patients receiving ibrutinib should be screened and receive prophylaxis (86).

Given the increased risk of second malignancies, the expert panel recommends routine cancer screening as part of ongoing care. This should include a referral of patients for dermatology examinations at a frequency of once a year (Recommendation 9.5). This reflects the higher risk of melanoma and non-melanomatous skin cancers seen in this group (87). Appropriate skin protection measures should also be recommended for these patients (6).

## Conclusion

This expert consensus guideline presents an up-to-date, evidence-based approach to the diagnosis and management of CLL, complementing the iwCLL guidelines by providing region-specific guidance tailored to the Saudi healthcare context, including local therapy availability, sequencing of novel agents, and practical management considerations. It aligns with current international recommendations while reflecting locally available resources, regulatory approvals, and clinical expertise. Over the past decade, CLL treatment has evolved significantly, offering patients a broad spectrum of highly effective therapeutic options. It is imperative that physicians remain informed of these advances and apply them judiciously. Nonetheless, important challenges persist–particularly in selecting and sequencing therapies, and in managing treatment-related toxicities. Ongoing evidence reviews, multidisciplinary collaboration, and real-world data integration will be critical to further improving outcomes for patients with CLL in Saudi Arabia.

## Data Availability

The original contributions presented in this study are included in this article/supplementary material, further inquiries can be directed to the corresponding author.
