# Supplementary material for: Clinical practice guidelines for the diagnosis and management of chronic lymphocytic leukemia in Saudi Arabia: consensus statement by an expert panel
Source: Front Med (Lausanne). 2026 Jan 14;12:1719364. doi: 10.3389/fmed.2025.1719364 (PMC12847048; doi:10.3389/fmed.2025.1719364)
Supplement: Supplementary file 1 [file Table_1.docx]

**Supplement Table 1. Consensus statements and degree of expert consensus (% of expert panel voting ‘agree’ or ‘strongly agree’) after two rounds of voting**

| **Section** | **Statement** | **Degree of consensus** | **Strength of recommendation** |
| --- | --- | --- | --- |
| **DIAGNOSIS AND STAGING OF CLL** | | | |
| 1. Diagnosis | 1.1 Diagnosis of CLL requires the presence of ≥5 x 10^9^/L clonal B lymphocytes in the peripheral blood, sustained for at least 3 months. | 100% | Strong |
|  | 1.2 The clonality of B lymphocytes should be confirmed by demonstrating immunoglobulin light chain restriction using flow cytometry. A panel of CD19, CD5, CD20, CD23, κ, and λ is usually sufficient to establish the diagnosis. | 100% | Strong |
|  | 1.3 Molecular genetics, mutational status of IGHV and variable heavy stereotypes, immunophenotypic markers, serum markers and marrow examination are not essential to diagnose CLL, but are important to predict the prognosis and/or assess the tumor burden. | 100% | Strong |
| 2. Staging | 2.1 The modified Rai classification defines low-risk disease as lymphocytosis with leukemia cells in the blood and/or marrow. Patients with peripheral blood lymphocytosis, enlarged lymph nodes in any site, and splenomegaly and/or hepatomegaly (lymph nodes being palpable or not) are defined as having intermediate-risk disease. Patients with disease-related anemia or thrombocytopenia (regardless of presence or absence of above features) are categorized as having high-risk disease. | 100% | Strong |
|  | 2.2 The Binet staging system is based on the number of involved lymphoid areas (defined by the presence of enlarged lymph nodes ≥1 cm in diameter or organomegaly) and presence of anemia or thrombocytopenia. Areas of involvement considered for staging include: head and neck, including the Waldeyer ring, axillae, groins, including superficial femorals, palpable spleen, and palpable liver. Stage A is classified as Hb ≥10 g/dL and platelets ≥100 x 109/L and ≤2 areas involved. Stage B is defined as Hb ≥10 g/dL and platelets ≥100 x 109/L and ≥3 areas involved. Stage C is considered as Hb <10 g/dL and/or a platelet count <100 x 109/L. | 100% | Strong |
|  | 2.3 The CLL-IPI includes clinical stage, age, IGHV mutational status, serum beta 2-microglobulin, and presence of del17p and/or TP53 mutations and can be used to identify high-risk patients. | 100% | Strong |
|  | 2.4 The CIRS may be used to assess comorbidities in CLL, although other scores to identify unfit patients are available. | 87.5% | Strong |
| **FIRST-LINE TREATMENT OF CLL** | | | |
| 3. Early-stage asymptomatic patients | 3.1 A ‘watch and wait’ strategy is recommended in patients with early asymptomatic disease. | 100% | Strong |
| 4. Symptomatic patients who are young and fit without del17p or TP53 mutations | 4.1 Chemotherapy is no longer recommended, given the overall survival benefit with ibrutinib + rituximab versus FCR in the E1912 trial and the risk of secondary malignancies with FCR. | 100% | Strong |
|  | 4.2 BTKi monotherapy is one of the options for patients with CLL without del17p mutation or TP53 aberrations who require treatment. | 100% | Strong |
|  | 4.3 Venetoclax + obinutuzumab is recommended as a time-limited therapy. | 87.5% | Strong |
|  | 4.4 Ibrutinib + venetoclax is an alternative fixed duration treatment option in patients aged under 60 years with no underlying comorbidities and who are willing to undergo therapy. | 100% | Strong |
|  | 4.5 MRD assessment is not typically used in clinical practice, but MRD-guided therapy may become a feasible future strategy. | 100% | Strong |
| 5. Symptomatic patients with del17p, TP53 mutations, or both | 5.1 Continuous BTKi therapy is recommended in patients with CLL who have del17p and/or TP53 aberrations. | 100% | Strong |
|  | 5.2 Venetoclax–obinutuzumab is one of the options for patients who prefer a time-limited therapy. | 100% | Strong |
|  | *5.3* Venetoclax-acalabrutinib (preferred) or venetoclax-ibrutinib (less preferred) are other time-limited treatment options for these patients. | 100% | Strong |
| 6. Symptomatic patients who are older and/or unfit without del17p or TP53 mutations | 6.1 Venetoclax + obinutuzumab is recommended as a time-limited therapy for these patients. | 87.5% | Strong |
|  | 6.2 BTK inhibitors (ibrutinib, acalabrutinib, and zanubrutinib) are recommended as continuous therapy. | 100% | Strong |
|  | 6.3 Second-generation BTKis (e.g. acalabrutinib and zanubrutinib) have a more favourable toxicity profile in patients with comorbidities, but ibrutinib remains a reasonable option given the availability of long-term data and the experience of physicians in managing therapy. | 100% | Strong |
|  | 6.4 There is no added benefit of rituximab in combination with BTKis. | 87.5% | Strong |
|  | 6.5 Obinutuzumab combined with BTKis is associated with additional benefit. | 62.5% | Weak |
|  | 6.6 Chlorambucil + obinutuzumab is a not a preferred option in patients who are not fit for therapy. | 75% | Strong |
| **TREATMENT OF RELAPSED/REFRACTORY CLL** | | | |
| 7. Relapsed/refractory disease | 7.1 Chemoimmunotherapy is not recommended for relapsed/refractory (R/R) CLL. | 75% | Strong |
|  | 7.2 BTKis or venetoclax in combination with rituximab are recommended in R/R disease regardless of mutation status, depending on what first-line regimen the patient received. | 87.5% | Strong |
|  | 7.3 BTKis may be preferred over venetoclax with rituximab in patients with TP53 aberrations. | 100% | Strong |
|  | 7.4 A BTKi in combination with venetoclax may be considered in all patients with R/R disease regardless of mutation status. | 87.5% | Strong |
|  | 7.5 If therapy is sequenced, venetoclax is preferred after a BTKi. | 87.5% | Strong |
|  | 7.6 Pirtubrutinib should be considered in patients refractory to a covalent BTKi and a BCL2i. | 100% | Strong |
|  | 7.7 PI3K inhibitors have efficacy in R/R CLL, but their value is limited by their safety profile. | 100% | Strong |
|  | 7.8 Clinical trials are recommended for patients with R/R CLL who are refractory to both BTK and BCL2 inhibitors. | 87.5% | Strong |
|  | 7.9 Beyond two lines of therapy, CAR T-cell therapy should be considered in patients with rapidly progressing disease. | 87.5% | Strong |
| 8. HSCT | 8.1 Allogeneic HSCT from a matched related or unrelated donor can be considered in young, fit patients with high-risk CLL who have failed at least one pathway inhibitor. | 62.5% | Weak |
|  | 8.2 Patients should be referred to a transplant center for consultation once their CLL has proven refractory to at least one pathway inhibitor. | 87.5% | Weak |
|  | 8.3 Older patients should generally not be considered for allogeneic HSCT due to the high mortality rate. | 87.5% | Strong |
| SUPPORTIVE CARE | | | |
| 9. Supportive care | 9.1 Patients with CLL should receive annual influenza vaccination, and COVID-19, pneumococcal, and Herpes zoster immunizations according to recommended schedules. Live vaccines should not be given during periods of immunosuppression from chemotherapy or immunotherapy. | 87.5% | Strong |
|  | 9.2 Antimicrobial prophylaxis requirements vary by therapeutic regimen, but vigilant observation and early initiation of treatment are warranted if symptoms develop. | 100% | Strong |
|  | 9.3 Use of prophylactic IVIG is reserved for patients experiencing recurrent serious infections. | 100% | Strong |
|  | 9.4 CLL patients should be screened for HBV prior to starting therapy with anti-CD20 mAb-containing regimens, idelalisib, and purine analogs. (STRONG) HBV carriers should start prophylactic antiviral therapy with entecavir before initiating these treatments. | 100% | Strong |
|  | 9.5 Consider referring patients for a dermatology examination once a year. | 62.5% | Weak |

Abbreviations: BTKi, Bruton’s tyrosine kinase inhibitor; CIRS, cumulative Illness Rating Scale; CLL, chronic lymphocytic leukemia; CLL-IPI, CLL International Prognostic Index; FCR, fludarabine, cyclophosphamide, rituximab; Hb, hemoglobin; HSCT, hemopoietic stem cell transplant; IGHV, immunoglobulin heavy chain; IVIG, intravenous immunoglobulin; MRD, minimal residual disease; PI3K, phosphoinositide 3-kinase; HBV, hepatitis B virus; mAb, monoclonal antibody
